# Highly selective and sensitive macrocycle-based dinuclear foldamer for fluorometric and colorimetric sensing of citrate in water

**DOI:** 10.1038/s41598-017-18322-w

**Published:** 2018-01-10

**Authors:** Md Mhahabubur Rhaman, Mohammad H. Hasan, Azmain Alamgir, Lihua Xu, Douglas R. Powell, Bryan M. Wong, Ritesh Tandon, Md. Alamgir Hossain

**Affiliations:** 1 Department of Chemistry and Biochemistry, Jackson State University, Jackson, MS 39217 USA; 2 Department of Microbiology and Immunology, University of Mississippi Medical Center, Jackson, MS 39216 USA; 3 Department of Chemical & Environmental Engineering and Materials Science & Engineering Program, University of California-Riverside, Riverside, CA 92521 USA; 4 Department of Chemistry and Biochemistry, University of Oklahoma, Norman, OK 73019 USA

## Abstract

The selective detection of citrate anions is essential for various biological functions in living systems. A quantitative assessment of citrate is required for the diagnosis of various diseases in the human body; however, it is extremely challenging to develop efficient fluorescence and color-detecting molecular probes for sensing citrate in water. Herein, we report a macrocycle-based dinuclear foldamer (**1**) assembled with eosin Y (EY) that has been studied for anion binding by fluorescence and colorimetric techniques in water at neutral pH. Results from the fluorescence titrations reveal that the **1**·EY ensemble strongly binds citrate anions, showing remarkable selectivity over a wide range of inorganic and carboxylate anions. The addition of citrate anions to the **1**·EY adduct led to a large fluorescence enhancement, displaying a detectable color change under both visible and UV light in water up to 2 μmol. The biocompatibility of **1**·EY as an intracellular carrier in a biological system was evaluated on primary human foreskin fibroblast (HF) cells, showing an excellent cell viability. The strong binding properties of the ensemble allow it to be used as a highly sensitive, detective probe for biologically relevant citrate anions in various applications.

## Introduction

The selective sensing of anions is an important area in supramolecular chemistry due to their significant roles in diverse chemical, biological, medicinal, and environmental science applications^1^. In particular, there is a growing interest in designing artificial receptors that can selectively recognize anions and act as sensors^2,3^. A wide variety of host receptors have been reported that can effectively bind anions in solution and in the solid states, exhibiting selectivity toward certain anions^4–7^. In this regard, carboxylates are of great interest due to their important roles in chemistry and biology^8–10^. Several multi-carboxylates including citrate, succinate, malate, and fumarate are critical chemical species in living cells that are involved in the *Krebs cycle* to generate energy used by aerobic cells in humans^11^. Other carboxylates such as oxalate, tartrate, and glutamate are produced as intermediates during the process of cellular metabolism^12,13^. An oxalate anion is naturally present in several foods and serves as a nutrient in the human body; however, an excess consumption of oxalate is associated with the development of kidney stones^14^. In particular, citrate is widely used in the pharmaceutical industry as an anticoagulant to stop blood clotting^15^ and in the food industry as a preservative across the broad spectrum of food and beverage products^16^. Since citrate in urine is considered to inhibit the crystallization of calcium salt, a low amount of citrate in urine is associated with the increased risk of urological diseases such as *nephrolithiasis* and *hypocitraturia* ^17,18^. Therefore, citrate is quantitatively determined by a number of instrumental methods, such as gas chromatography, high-performance liquid chromatography, capillary electrophoresis, and enzyme assay^19–21^ that are limited to *in vitro* samples. In this regard, molecular sensors, which are based on non-covalent interactions, can provide real-time information of intracellular chemistry^22^. As a result, the development of simple and color-detecting molecular devices to identify citrate is highly desirable, particularly in water at neutral pH.

Many artificial chemosensors have been developed for the detection of citrates in recent years^23–26^. For example, Anslyn and co-workers developed a *tris*-guanidinium-based chemosensor that effectively binds citrate in aqueous solution^26^. Schmuck and Schwegmann synthesized a tripodal guanidininocarbonyl pyrrole as a citrate binding host^27^. Stang, Chi, and coworkers reported ruthenium-based tetranuclear metalla-bowls that bind with multi-carboxylate anions such as oxalate, tartrate, and citrate^28^. The majority of reported receptors are designed based on a covalently attached signaling unit (chromophore or fluorophore), that are primarily based on electrostatic or hydrogen bonding interactions. Recently, dinuclear metal complexes were shown as excellent hosts that recognize certain anions through metal-ligand interactions^29–42^. Nelson and coworkers reported a dicopper(II) complex of a *m*-xylyl-based cryptand for N_3_^−^ or NCS^−^^31^. Fabbrizzi and coworkers studied a dicopper(II) complex of a furan-based cryptand for halides^32^, and a dinuclear copper(II) complex of an expanded cryptand for nucleoside monophosphates^33^. Delgado and coworkers described dinuclear copper(II) complexes for binding of oxalate and succinate anions^35,36^. To the best of our knowledge, dinuclear metal compounds, however, have not been exploited for citrate binding so far. Herein, we report a macrocycle-based dinuclear copper(II) complex (**1**) containing a folded cavity that selectively binds citrate over a wide range of anions in pure water at neutral pH, providing a sharp color change in the presence of an external dye (eosin Y).

## Results and Discussion

### Design and Synthesis

**Figure 1:**
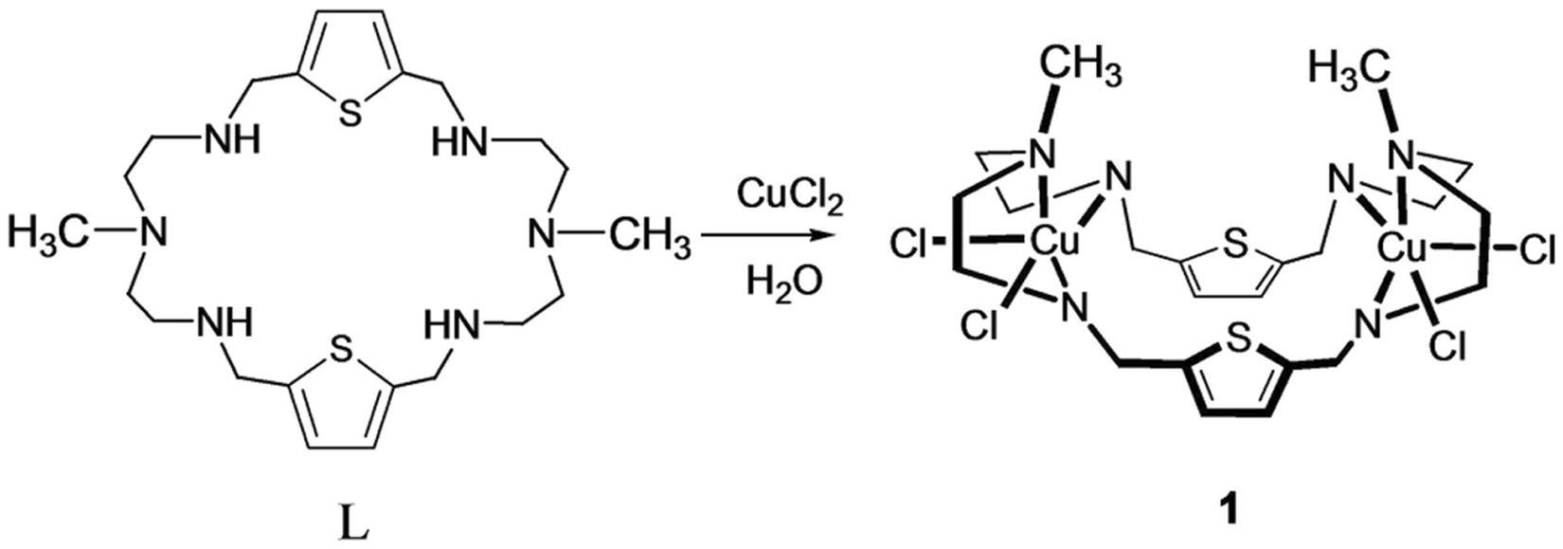
Macrocycle **L** and its dinuclear copper complex **1**.

The design of the macrocycle-based dimetallic complex **1** was based on a concept developed by Lehn, who reported a *bis*tren-based copper cryptate termed as a “cascade complex” for bridging of an anion between the two metal centers^43^. This concept was successfully applied to other *bis*tren-cryptands^31–34^ and to *bis*dien-based macrocycles^36–42,44^ incorporated with two transition metal ions (e.g. Cu^II^). In this study, we have chosen a simple methyl-substituted hexaaza-macrocycle **L** (Fig. 1) that was synthesized from the high dilution condensation reactions of an equimolar ratio of *N*-methyl-2,2′-diaminodiethylamine and 2,5-thiophenedicarbaldehyde followed by the reduction with NaBH_4_ in methanol^45^. The dinuclear copper(II) complex [Cu_2_^II^(**L**)Cl_4_] (**1**) was prepared by adding two equiv. of copper(II) chloride to **L** in a water-methanol mixture. The blue micro-crystals that formed immediately were filtered and recrystallized from slow evaporation of a water solution, yielding X-ray quality crystals. Numerous attempts to grow crystals of anion complexes were unsuccessful. Through the analysis from the indicator displacement assay (IDA), we have shown that the receptor binds the citrate anion selectively over a wide range of dicarboxylates and inorganic anions, forming a 1:1 stoichiometric complex. High level DFT calculations further support the formation of the citrate complex with the receptor through metal-ligand coordination.

### Crystal Structure Analysis

**Figure 2:**
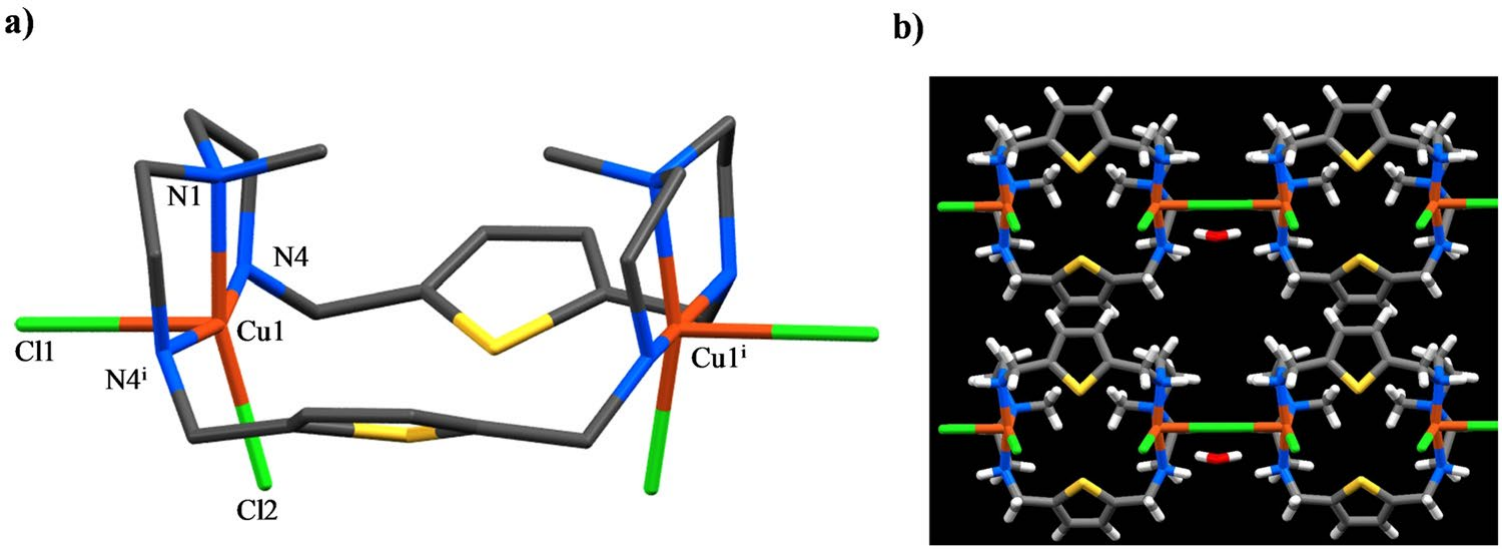
(**a**) Side view of the [Cu_2_^II^(**L**)Cl_4_] motif in the crystal structure of [Cu_2_^II^(**L**)Cl_3_]_n_∙nCl∙xH_2_O: Hydrogen atoms are not shown for clarity. (**b**) Crystal packing as viewed along the *a*-axis.

The X-ray analysis of the copper complex reveals that it crystallizes as [Cu_2_^II^(**L**)Cl_3_]_n_∙nCl∙xH_2_O, (**1**′) in the orthorhombic space group *Amm2* with two copper(II) ions at both *N*_*3*_ sites. Each copper ion is coordinated with three macrocyclic nitrogens and two chlorides forming a square pyramidal geometry. As shown in Fig. 2a, the macrocycle is folded to form a boat-like shape with an empty cavity where the Cu···Cu distance is 6.3071(11) Å. A similar structure was observed previously for [Cu_2_^II^(**L**)Br_4_]·2H_2_O, with a Cu···Cu distance of 6.243 Å^40^. Each coordinating copper contains one equatorial and one axial chloride. The Cu−Cl_equatorial_ distance is 2.2416(12) Å for Cu1−Cl1, while the Cu−Cl_axial_ distance is 2.7191(5) Å for Cu1−Cl2. The longer distance in Cu−Cl_axial_ is due to a characteristic Jahn-Teller distortion^39^. The Cu−N distances range from 2.022(2) to 2.034(4) Å. The bond angle of N4−Cu1−N1 (or N4^i^−Cu1−N1) is 84.85(7)°, while that of N4−Cu1−Cl1 (or N4^i^−Cu1−Cl1) is 96.65(7), which are almost close to 90° required for a perfect square pyramidal geometry. The chloride Cl3 (not shown in Fig. 2) is disordered over three sites on a mirror with refined occupancies of 0.480(5), 0.287(7), and 0.234(6). It is assumed that the molecule in its solid state as [Cu_2_^II^(**L**)Cl_3_]_n_∙nCl∙xH_2_O would provide a charge-balanced formula as [Cu_2_^II^(**L**)Cl_4_], (1) in solution which was used for the solution binding studies. The macrocycles are connected through the axial chloride to form an infinite polymeric chain along the *c*-axis, sitting on a crystallographic site of *mm* symmetry. Furthermore, each chain is linked with a neighboring chain through the CH···π interactions of two aromatic groups, providing a sheet-like structure (Fig. 2b).

### Fluorescence Binding Studies Based on IDA Mechanism

**Figure 3:**
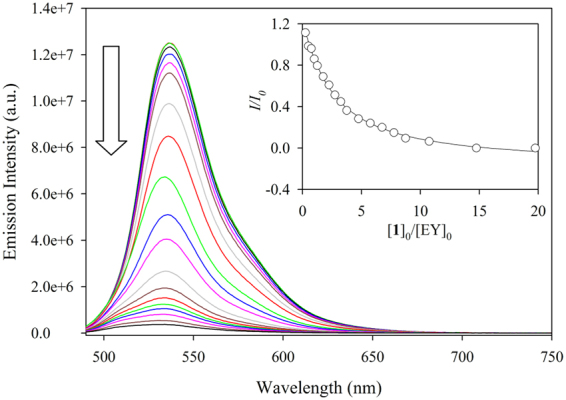
The quenching of fluorescence intensity of EY upon the gradual addition of **1** in water at pH 7.0 [λ_ex_ = 470 nm, λ_em_ = 536 nm]. The inset shows the titration plot of *I*/*I*_0_ against [**1**]_0_/[EY]_0_ at λ_em_ = 536 nm.

An indicator displacement assay (IDA) was introduced by Anslyn in his elegant work on anion receptors for sensing of citrate in beverages^26,46^. In this case, a negatively charged fluorescent dye (5-carboxyfluorescein) was employed to bind to a receptor consisting of three guanidinium groups, forming an ensemble through charge pairing and/or hydrogen bonding interactions. Upon the addition of citrate, the dye was removed from the ensemble due to the stronger interaction with the receptor, leading to a significant absorption change. Previous studies showed that such a principle can be exploited for dinuclear transition metal complexes to examine their binding selectivities for certain anions through metal-ligand bonding interactions^33,40^. In our study, we applied the IDA mechanism to study the anion binding ability of **1** employing a commercially available external fluorescent dye, eosin Y (EY). As shown in Fig. 3, the fluorescence intensity of EY gradually decreased upon the increasing addition of **1** (2.0 × 10^−4^ M) to a solution of EY (2.0 × 10^−6^ M), resulting in an almost complete quenching of the emission. The change in the fluorescence intensity (*I/I*_*o*_) provided the best fit to a 1:1 binding model^45^, yielding a binding constant (*K*) of 2.51 × 10^5^ M^−1^. Such a quenching is due to the ion-pair formation (**1**·EY) of the negatively-charged dye with the metal complex that quenches the excited state of EY through the electron/energy-transfer process^35^. Hence, the adduct **1**·EY could be used as a “fluorescent OFF-ON” probe for optical as well as visual detection of biologically important anions through the restoration of the fluorescence of EY released to the solution.

**Figure 4:**
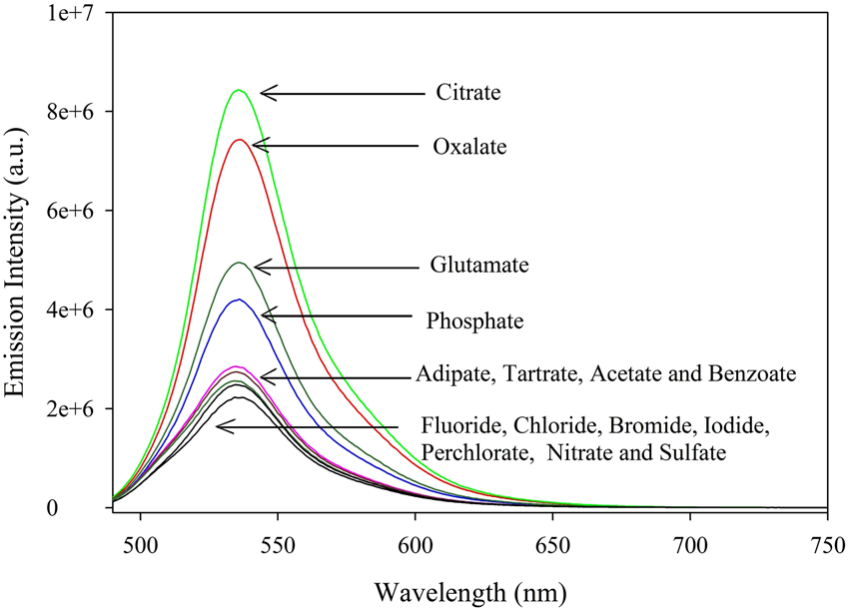
The change in fluorescence intensity of **1**·EY (**1**/EY = 5:1) upon addition of 5 equiv. anions in water at pH 7.0 [λ_ex_ = 470 nm, λ_em_ = 536 nm].

The **1**·EY adduct was used as a fluorescence probe for a number of anions, including inorganic halides (fluoride, chloride, bromide, and iodide), oxoanions (nitrate, sulfate, perchlorate, and phosphate), and carboxylates (citrate, oxalate, glutamate, adipate, tartrate, benzoate, and acetate). As shown in Fig. 4, the addition of different anions (5 equiv.) to the **1**·EY ensemble resulted in the highest restoration of the fluorescence for citrate followed by oxalate, glutamate, and phosphate. When other carboxylates such as adipate, tartrate, benzoate, and acetate were added to the solution, only a weak enhancement of the fluorescence intensity was observed, indicating that those anions interact weakly with **1**·EY. This observation implies that the host provided binding sites for selected anions through metal-ligand interactions with varying strength, releasing different amounts of the dye to the solution, as signaled by the fluorescence enhancement. In contrast, the fluorescence of the **1**·EY ensemble was unchanged for other inorganic anions including fluoride, chloride, bromide, iodide, nitrate, sulfate, and perchlorate. This result suggests that the citrate is the strongest candidate among all investigated anions to displace the dye from the dinuclear copper(II) ensemble due to the formation of [**1**·citrate] complex in water.

**Figure 5:**
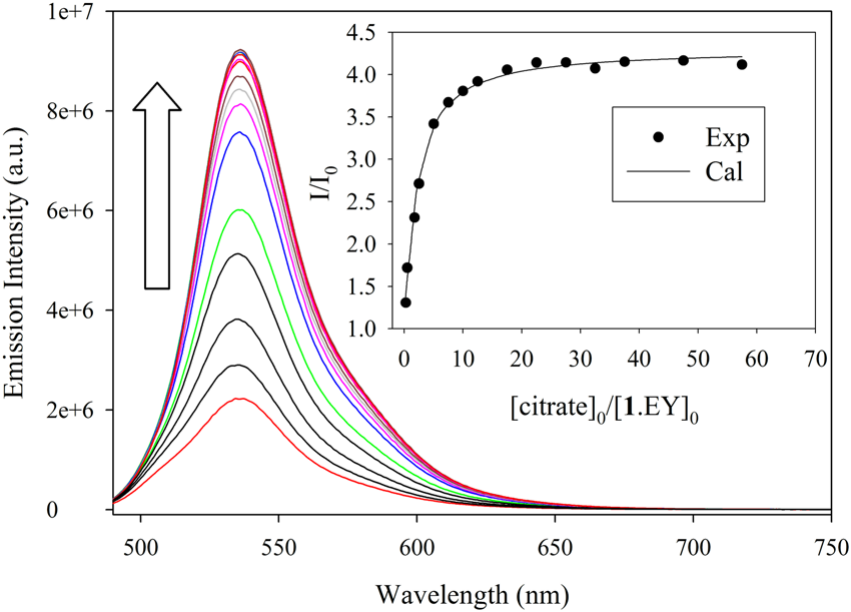
The enhancement of fluorescence intensity of [**1**·EY] (**1**/EY = 5:1, [EY]_0_ = 2.0 × 10^−6^ M) upon the addition of citrate in water at pH 7.0 [λ_ex_ = 470 nm, λ_em_ = 536 nm]. The inset shows the titration plot of *I*/*I*_0_ against [citrate]_0_/[**1**·EY]_0_ at λ_em_ = 536 nm.

The binding affinity of **1**·EY for citrate was examined from the titration of **1**·EY with an incremental addition of citrate. Figure 5 shows the gradual enhancement of the fluorescence after the addition of citrate. As determined from the Job’s plot, a 1:1 binding was observed (Figure S13 in Supporting Information). Analysis of the binding isotherm from the fitting of *I*/*I*_0_ with the ratio of [citrate]_0_/[**1**·EY]_0_ gave the best fit to a 1:1 binding model^47^, providing a conditional binding constant of *K* = 6.5 × 10^5^ M^−1^ for the equilibrium of **1**·EY + citrate = **1**·citrate + EY. This value is higher than the binding constant of **1** with EY (*K* = 2.51 × 10^5^ M^−1^), making the **1**·EY ensemble ideal for citrate anion. The observed stability for the [**1**·citrate] complex is higher than 7.94 × 10^4^ M^−1^ found for guanidiniocarbonyl pyrroles^27^, and comparable to 3.9 × 10^5^ M^−1^ for a cyclam-based Cu(II) complex^48^ in water. The conditional binding constants were also determined for other anions including oxalate, glutamate, adipate, tartrate, benzoate, acetate, and phosphate by fluorescence titration experiments of **1**·EY with their potassium salts in water at pH 7.0 under identical conditions, which also provided the best fit for a 1:1 binding of host/guest (Figures S5–S8). The results, as displayed in Table 1, suggest that the new fluorescence probe provides the highest affinity for tribasic citrate among all the anions studied. Other anions including oxalate and glutamate also showed high binding to **1**·EY, with binding constants of 1.9 x.10^5^ and 1.0 x.10^5^ M^−1^, respectively. Moderate binding is observed in the case of phosphate and adipate. The **1**·EY ensemble shows the binding affinity for carboxylates in the order of citrate > oxalate > glutamate > adipate > tartrate > acetate > benzoate. However, with the exception for phosphate, no binding is observed for halides and oxoanions to **1**·EY.

**Table 1:** Conditional constants (*K*) for various anions for the equilibria: **1**·EY + Anion = **1**·Anion + EY, as measured by the indicator displacement assay in water at pH 7.0.

Anion	*K, M* ^−1a^
Citrate	6.5 × 10^5^
Oxalate	1.9 × 10^5^
Glutamate	1.0 × 10^5^
Phosphate	3.9 × 10^4^
Adipate	1.6 × 10^4^
Tartrate	7.9 × 10^3^
Acetate	1.9 × 10^3^
Benzoate	3.9 × 10^2^

^a^Estimated deviations are less than 15% (based on the standard deviation from the fit of experimental values).

**Figure 6:**
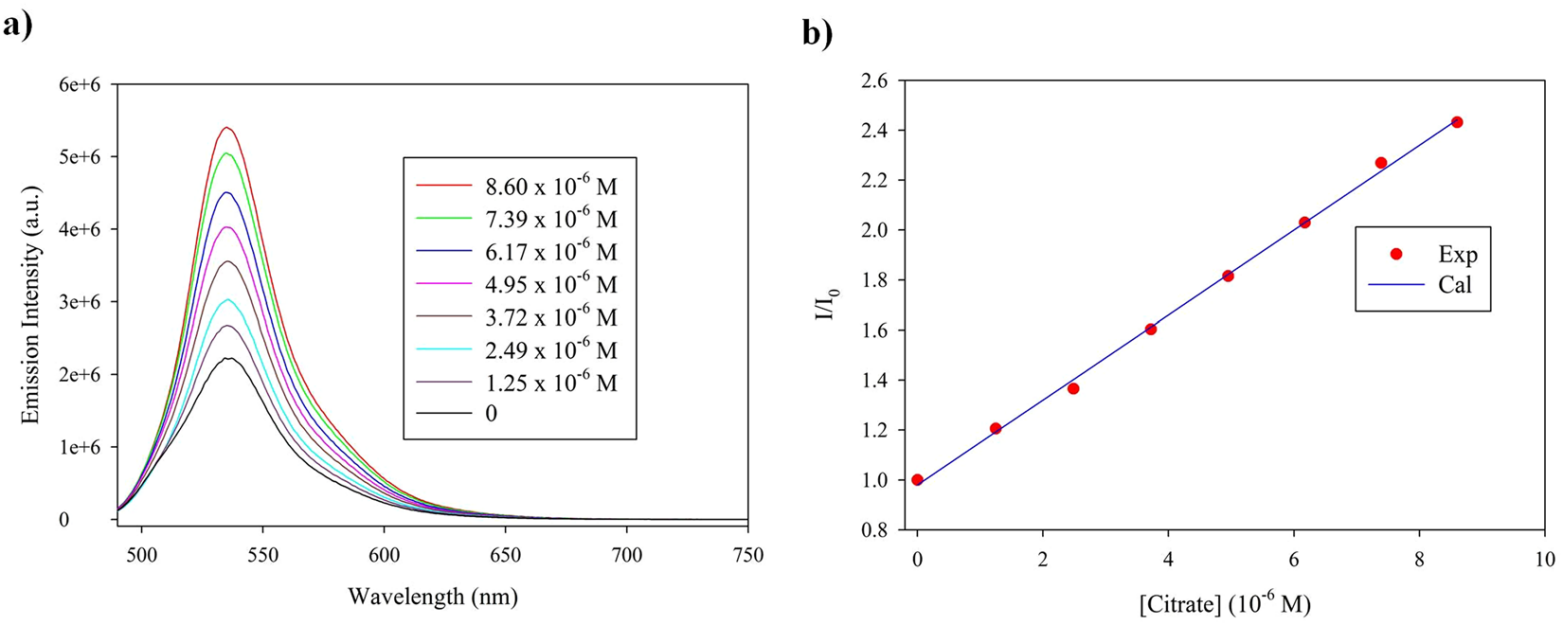
Determination of the detection limit for citrate: (**a**) The enhancement of fluorescence intensity of [**1**·EY] (**1**/EY = 5:1, [EY]_0_ = 2.0 × 10^−6^ M) upon the addition of citrate in the range of 1.25 × 10^−6^ to 8.60 × 10^−6^ M^−1^; (**b**) Citrate detection scale as plotted with *I/I*_*o*_ against the concentration of citrate [λ_ex_ = 470 nm, λ_em_ = 536 nm].

The detection limit for citrate was evaluated by titrations of **1**·EY (**1**/EY = 5:1, [EY]_0_ = 2.0 × 10^−6^ M) with an incremental addition of citrate (5.0 × 10^−4^ M) in water at pH 7. As shown in Fig. 6, the fluorescence ratio (*I/I*_0_) shows a good linear dependency with citrate in the range of 1.25 × 10^−6^ to 8.60 × 10^−6^ M (*R* ^*2*^ = 0.9991). The linear regression of the fluorescence change provides the equation: *y* = 0.1700*x* + 0.9803 (*R* ^*2*^ = 0.9979), allowing us to estimate the lowest detection limit up to 0.45 ± 0.02 μM^49^. A lower detection limit (0.18 ± 0.01 μM) of citrate was recently reported by Yen and coworkers with a thiourea-based cleft; however, it was in a solution of DMSO/H_2_O (4/1, v/v)^50^.

### Colorimetric Studies

**Figure 7:**
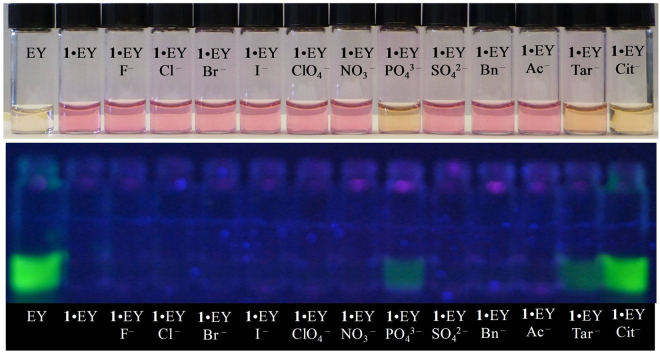
Colorimetric detection of citrate against various anions with **1**·EY (**1**/EY = 5:1, [EY]_0_ = 2 × 10^−5^ M) in water at pH 7.0. Top: visible light; Bottom: UV light at 365 nm. 10 equiv. of anion were added to the solution of **1**·EY.

**Figure 8:**
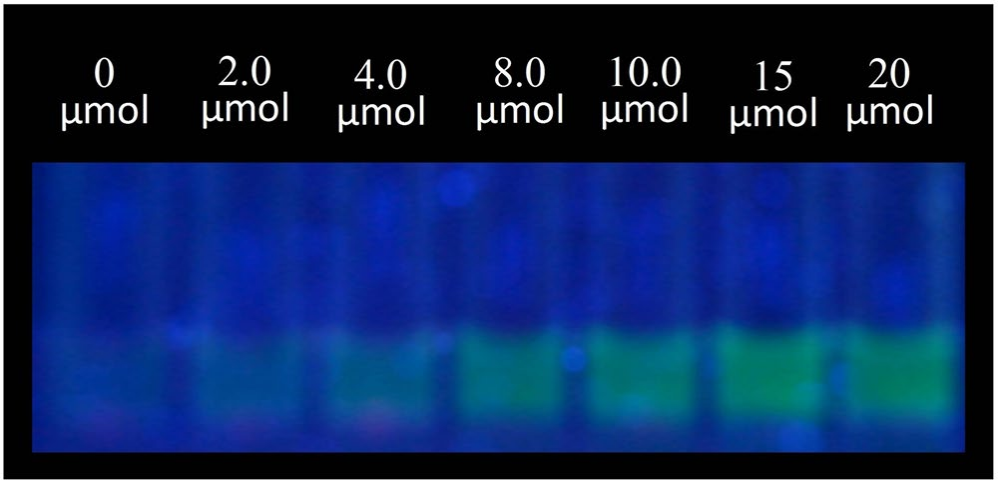
Progressive color change with an increasing amount of citrate in the range of 0 to 20 μmol to [**1**·EY] (**1**/EY = 5:1, [EY]_0_ = 4 × 10^−6^ M) in water at pH 7.0 under UV light at 365 nm.

To evaluate the color-detecting ability of **1**·EY for anions, the ensemble was mixed with 10 equiv. of different anions in water buffered with 20 mM HEPES at pH 7.0, and their colors were examined under both visible and UV light. As shown in Fig. 7, the addition of citrate to **1**·EY resulted in a noticeable color change under visible (top) light as well as under UV light at 365 nm (bottom). Under visible light, the deep magenta color of **1**·EY solution changed to pale orange due to the addition of citrate, tartrate, and phosphate. It is important to note that the color change was very distinct (pale purple to green yellow) for citrate under the UV light at 365 nm, showing almost complete restoration of the original color of EY. For phosphate and tartrate, the color change was still noticeable but with less fluorescence, agreeing with the binding constants measured from fluorescence titrations. Furthermore, the concentration dependent colorimetric titrations, as shown in Fig. 8, suggest that citrate can be detected in water as low as 2 μmol from the color change under the UV light.

### Cytotoxicity Assessment

**Figure 9:**
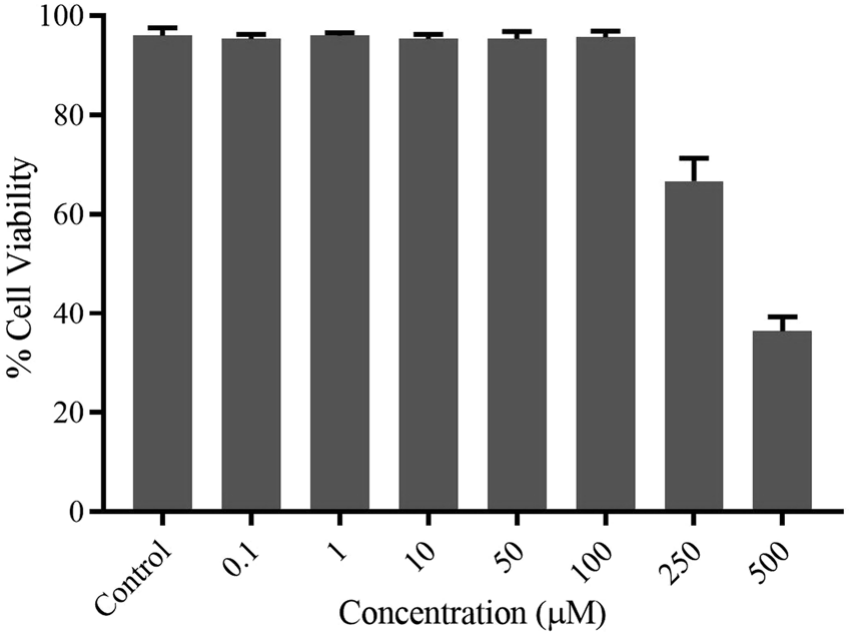
Viability of HF cells upon the treatment with **1**·EY. HF cells were mock treated (control) or treated with **1**·EY (0.1–500 µM) for 72 hours and cell viability was quantified using trypan blue exclusion assay. Error bars represent standard error of the mean from three independent experiments.

**Figure 10:**
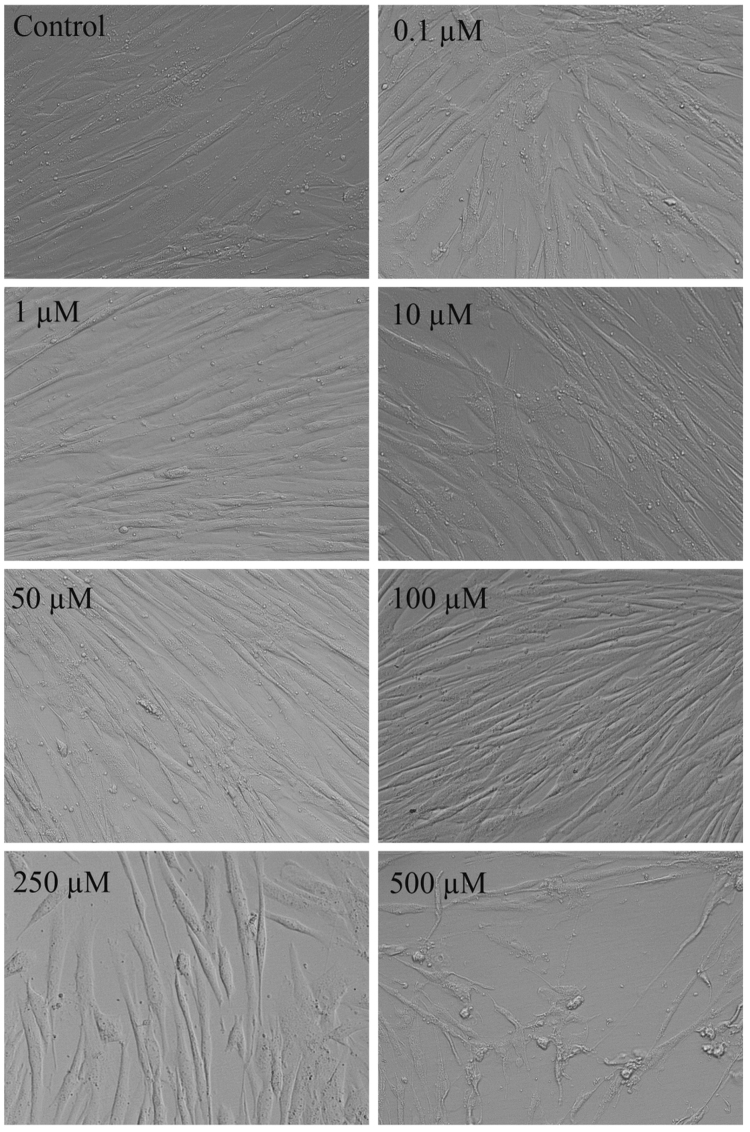
Bright-field images of HF cells upon the treatment with **1**·EY. HF cells were mock treated (control) or treated with **1**·EY (0.1–500 µM) for 72 hours and live cell images were captured.

To study the compatibility of **1**·EY as a sensor in a biological system, cytotoxicity was assessed on primary human foreskin fibroblast (HF) cells. These primary cells represent human tissue much better than any commercially available cell line. HF were treated with **1**·EY (0.1– 500 µM) for 72 hours and cell viability was quantified using trypan blue exclusion assay (Figs. 9 and 10)^51^. As shown in Fig. 9, the cell viability was near 100% for up to 100 µM concentration of **1**·EY. However, a significant cell cytotoxicity was observed at higher concentrations of **1**·EY (250 and 500 µM). Taken together, these data indicate excellent biocompatibility of **1**·EY in mammalian cells up to 100 µM concentration.

### Computational Studies

**Figure 11:**
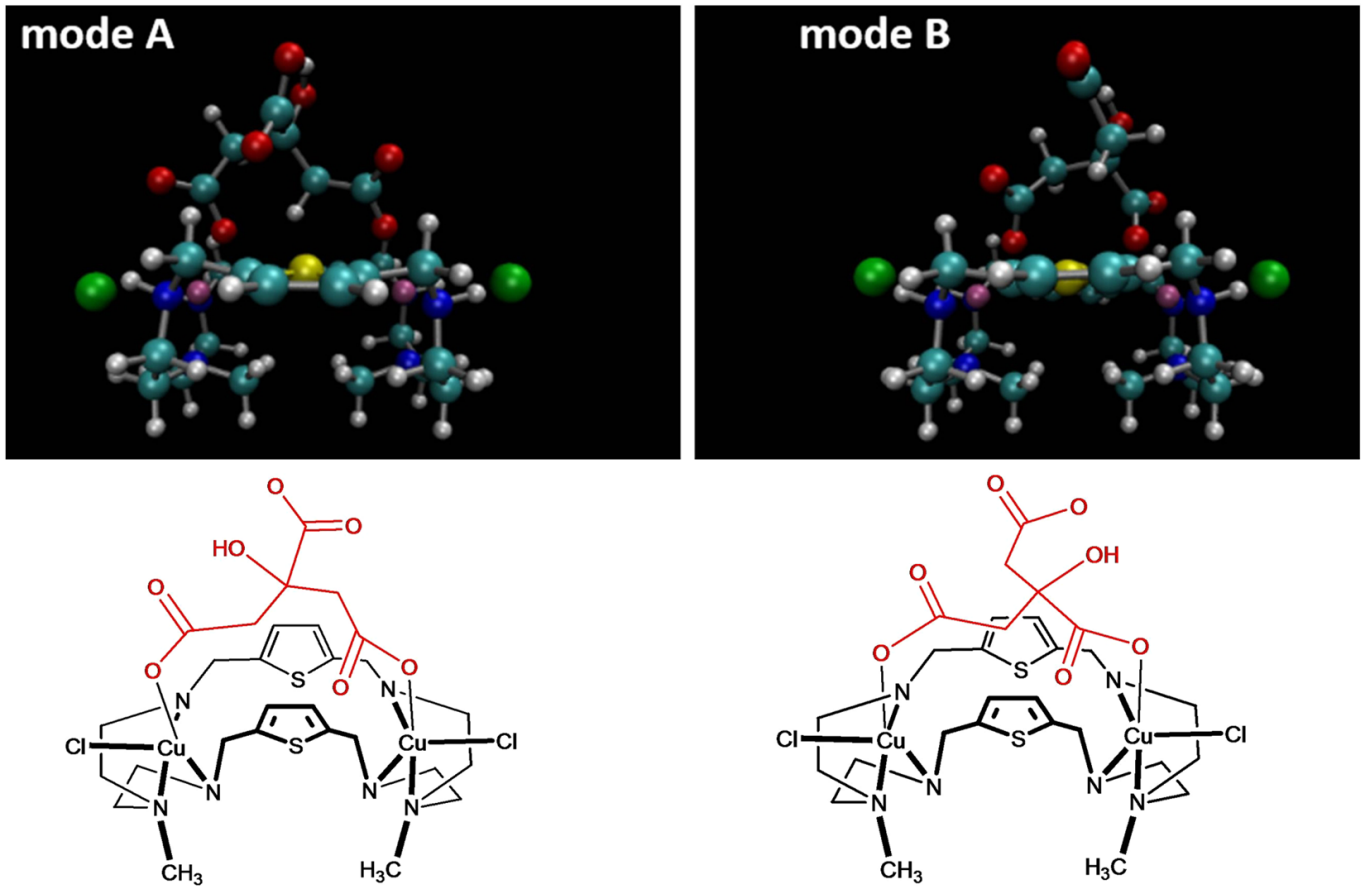
DFT-optimized geometries for two different binding configurations (represented by the chemical structures), denoted as mode A (left) and mode B (right), of the citrate complex with [Cu_2_^II^(**L**)Cl_2_]^2+^ carried out at the M06L/6-31 g(d,p) level of theory.

To shed additional mechanistic insight into the binding motifs of the dinuclear copper complex, we carried out a series of density functional theory (DFT) calculations using the M06L functional. We have specifically chosen the M06L functional for our studies due to its accuracy and widespread use in various organometallic compounds^52,53^ as well as noncovalent interactions for large systems^54^. An all-electron polarized 6–31 g(d, p) basis set was used for all geometry optimizations, and a larger 6–311 g(d, p) basis was used as a final single-point energy on the optimized geometry. Both the geometry optimizations and the final-single point energy calculations were carried out in the presence of a polarizable continuum water model (PCM). The specific PCM model used in this work is the implementation devised by Tomasi and co-workers^55^, that creates a solute cavity via a set of overlapping spheres to calculate the solvent reaction field. Fully unconstrained geometry optimizations were carried out on both the isolated receptor as [Cu_2_^II^(**L**)Cl_2_]^2+^ and various citrate-bound motifs. From these calculations, we found that the receptor formed a 1:1 complex with citrate, showing two different binding configurations: one with two symmetric carboxylate groups (mode A) and the other with two unsymmetric carboxylate groups (mode B), as shown in Fig. 11. The third unbound carboxylate group in each binding mode is H-bonded with the OH group of the citrate (OH···O = 2.533 and 2.620 Å in A and B modes, respectively). For each of the optimized geometries, the binding energy (ΔE) was calculated with a 6–311 g(d, p) basis as described earlier^41^, yielding an attractive ΔE of −70.79 kcal/mol for mode A and −73.07 kcal/mol for mode B. The most attractive binding energy for mode B reflects the preferred orientation of the citrate with the dinuclear copper complex in mode B.

## Conclusions

A macrocycle-based dinuclear foldamer has been used as an efficient probe in an approach of *Indicator Displacement Assay* for colorimetric and fluorescent sensing of citrate, which operates by a non-covalent binding principle involving metal-ligand interactions in pure water at neutral pH. The addition of citrate to the ensemble led to the complete restoration of the fluorescence (fluorescent-ON) of the dye due to the formation of the complex that was also supported by high level DFT calculations. This readily obtainable chemosensor has been shown to recognize citrate with strong sensitivity and selectivity over a wide range of anions in water, displaying a detectable color change under both visible and UV light in water up to 2 μmol. The synthesized ensemble is capable of sensing a citrate anion with the lowest detection limit up to 0.45 ± 0.02 μM in water, as calculated from the emission change. The dinuclear complex has been proven to be fully biocompatible in human foreskin fibroblast cells. The excellent anion binding properties in water and biocompatibility observed towards mammalian cells demonstrates that this chemosensor can be used as a potential sensing probe for the detection of biologically relevant citrate anions for various biological and chemical applications.

## Methods

### General

The chemicals used for this work were purchased from Sigma-Aldrich as reagent grade and used as received. Nuclear magnetic resonance (NMR) spectra were recorded at 25 °C on a Varian Unity INOVA 500 FT-NMR. Chemical shifts were measured in CDCl_3_ and calibrated against TMS as an external reference used in a sealed capillary tube. All NMR data were processed and analyzed with MestReNova Version 6.1.1-6384. The mass spectral data were obtained using an ESI-MS positive mode on a FINNIGAN LCQDUO. Elemental analysis was carried out using an ECS 4010 Analytical Platform (Costech Instrument) at Jackson State University. The fluorescence titrations were carried out using a Fluoromax-4 spectrofluorometer (HORIBA Scientific).

### Synthesis

***L*** *: N*-methyl-2,2′-diaminodiethylamine (0.6042 g, 5.1 × 10^−3^ mol) in CH_3_OH (300 mL) and 2,5-thiophenedicarboxyaldehyde (0.7218 g, 5.1 × 10^−3^ mol) in CH_3_OH (300 mL) were added simultaneously to a three-neck flask containing 600 mL of CH_3_OH over 4 h under stirring at 0 °C. The reaction mixture was further stirred overnight at room temperature. The solvent was removed under vacuum, and 100 mL of CH_3_OH was added to the residue. The imine that formed was reduced by NaBH_4_ (1.2 g) at room temperature overnight. The solvent was removed under vacuum, and the resulting reaction product was dispersed in water (100 mL). The aqueous phase was extracted by CH_2_Cl_2_ (3 × 100 mL). The organic portions were collected and dried by anhydrous MgSO_4_ (2g). The solid was filtered off, and the solvent was evaporated to dryness. The crude product was purified by column chromatography (neutral alumina, 2% CH_3_OH in CH_2_Cl_2_). Yield: 0.82 g, (62%). ^1^H NMR (500 MHz, CDCl_3_, TMS): *δ* 6.75 (s, 4 H, Ar*H*), 3.91 (s, 8 H, ArC*H*_2_), 2.71 (t, 8 H, *J* = 5.5 Hz, NCH_2_C*H*_2_), 2.48 (t, 8 H, *J* = 5.5 Hz, NC*H*_2_CH_2_), 2.14 (s, 6 H, NC*H*_3_), ^13^C NMR (125 MHz, CDCl_3_): *δ* 124.48 (Ar-C), 56.70 (Ar*C*H_2_), 48.64 (NCH_2_ *C*H_2_), 46.28 (N*C*H_2_CH_2_), 42.32 (N*C*H_3_). ESI-MS: *m*/*z* (+) 451.3 [M + H]^+^. Anal. Calcd. for C_22_H_36_N_6_S_2_: C, 58.63; H, 8.50; N, 18.65. Found: C, 58.60; H, 8.52; N, 18.48.

#### [Cu_2_^II^(**L**)Cl_4_]·H_2_O, (**1**)

The free amine **L** (100 mg, 0.222 mmol) was mixed with two equiv. of CuCl_2_·H_2_O (75.7 mg, 0.444 mmol) in 2 mL of H_2_O. The solution was stirred at 60 °C for one hour. The deep blue solid that appeared immediately was collected by filtration and washed by diethyl ether. The microcystals were recrystallized from slow evaporation of a water solution, yielding X-ray quality crystals. Yield: 150 mg, 88% yield. The compound was characterized by X-ray diffractometer. Anal. Calcd. for C_22_H_38_Cl_4_Cu_2_N_6_OS_2_: C, 35.92; H, 5.21, N, 11.42. Found: C, 35.85; H, 5.22; N, 11.44.

### Fluorescence Titration Studies

All fluorescence titrations were performed using a Fluoromax-4 spectrofluorometer (HORIBA Scientific) in water at pH 7.0. The pH was adjusted by using 0.02 M HEPES purchased from Sigma Aldrich Chemical Company. The experimental conditions were used as: λ_ex_ = 470 nm, λ_em_ = 536 nm, d_ex_ = 2, d_em_ = 5 for eosin Y dye.

#### Binding constant of **1** for EY

The binding affinity of **1** for EY for the formation of **1**·EY was determined from the titration of EY with **1** in water at pH 7.0. In this case, the quenching of fluorescence was observed after the addition of **1** to EY. Initial concentrations of EY and **1** were 2.0 × 10^−6^ M and 2.0 × 10^−4^ M, respectively. Each titration was performed by 15 measurements varying the [**1**]_0_/[dye]_0_ = 0–25, and the association constant (*K*) was calculated by fitting the ratio of *I/I*_*0*_ with a 1:1 association model using eq. (1).

1 $${\rm{\Delta }}{\rm \Phi}={({[{\bf{1}}]}_{0}+{[{\rm{EY}}]}_{0}+1/K-{({[{\bf{1}}]}_{0}+{[{\rm{EY}}]}_{0}+1/K)}^{2}-4{[{\rm{EY}}]}_{0}{[{\bf{1}}]}_{0})}_{1/2}){{\rm{\Delta }}{\rm{\Phi}}}_{{\rm{m}}{\rm{a}}{\rm{x}}}/2{[{\rm{EY}}]}_{0}$$

where Ф = *I/I*_*0*_, *I*_*0*_ = initial fluorescence intensity of EY, *I* = fluorescence intensity after the addition of **1**. The error limit in K was less than 15%.

#### Conditional constants of **1**·EY for anions

The binding affinity of **1**·EY for different anions was examined from the competition reaction, and the conditional constants were derived from the titration of **1**·EY with anions in water under the same experimental conditions described in the preceding section. In this case, EY was displaced by the addition of an anion, resulting in an enhancement of the fluorescence intensity. Initial concentrations of **1**·EY (**1**/EY = 5:1) and an anion were 2.0 × 10^−6^ M, and 2.0 × 10^−3^ M, respectively. Each titration was performed by 15 measurements varying the [anion]_0_/[**1**·EY]_0_ = 0–60, and the conditional constant (*K*) was calculated by fitting the ratio of *I/I*_*0*_ with a 1:1 association model using eq. (2).

2 $${\rm{\Delta }}{\rm \Phi}={({[{\rm{A}}]}_{0}+{[{\bf{1}}\cdot {\rm{EY}}]}_{0}+1/K-{({[{\rm{A}}]}_{0}+{[{\bf{1}}\cdot {\rm{EY}}]}_{0}+1/K)}^{2}-4{[{\bf{1}}\cdot {\rm{EY}}]}_{0}{[{\rm{A}}]}_{0})}_{1/2}){\rm{\Delta }}{\rm \Phi}_{{\rm{m}}{\rm{a}}{\rm{x}}}/2{[{\bf{1}}\cdot {\rm{EY}}]}_{0}$$

where, Ф = *I/I*_*0*_, A = anion, *I*_*0*_ = initial fluorescence intensity of **1**·EY, *I* = fluorescence intensity after the addition of an anion. The error limit in *K* was less than 15%.

#### Colorimetric studies

The colorimetric studies of the **1**·EY ensemble for different anions were performed under both visible and UV light (365 nm). Stock solutions of [**1**] = 2 × 10^−3^ M, [EY] = 2 × 10^−3^ M and [anion] = 2 × 10^−3^ M were prepared separately in water buffered with a 0.02 M HEPES solution at pH = 7.0. The **1**·EY ensemble was prepared by mixing **1** with EY at a ratio of 5:1. The **1**·EY ensemble was mixed with 10 equiv. of each anion, and the solution was further diluted to maintain the concentration of [EY] = 2 × 10^−5^ M. Each sample (1 mL) was transferred in a vial and the color was examined by naked eye under both a visible light and a UV lamp at 365 nm.

The colorimetric detection limit of citrate was evaluated using a 0.02 M HEPES solution in water at pH = 7.0. The stock solutions of **1** (2 × 10^−3^ M) was mixed with EY (2 × 10^−3^ M) at a ratio of 5:1 and the solution was further diluted to maintain a concentration of [EY] = 4 × 10^−6^ M. A stock solution of [citrate] = 2 × 10^−3^ M was prepared in water buffered with a 0.02 M HEPES solution. 0, 1.0, 2.0, 4.0, 5.0, 7.5, and 10.0 µL of citrate (2 × 10^−3^ M) were added separately to a vial containing 1.0 mL of **1**·EY ([**1**]/[EY] = 5:1, [EY] = 4 × 10^−6^ M). Final concentrations of the citrate solution were 0, 2.0, 4.0, 8.0, 10.0, 15.0, and 20 µM. The samples were then examined under both visible and UV light.

### X-ray Crystallography

Intensity data for **1**′ were collected using a diffractometer with a Bruker APEX ccd area detector^56^ and graphite-monochromated MoKα radiation (λ = 0.71073 Å). The sample was cooled to 100(2) K. Cell parameters were determined from a non-linear least squares fit of 5856 peaks in the range 2.42 < θ < 28.25. The orthorhombic space group *Amm*2 was determined by systematic absences and statistical tests and verified by subsequent refinement. The structure was solved by direct methods and refined by full-matrix least-squares methods on *F* ^2^ ^57^. The absolute structure was determined by refinement of the Flack parameter^58^. The polar axis restraints were taken from Flack and Schwarzenbach^59^. Crystal data: [Cu_2_^II^(C_22_H_36_N_6_S_2_)Cl_3_]_n_∙nCl∙xH_2_O, orthorhombic, *a* = 11.7449(10) Å, *b* = 16.8588(14) Å, *c* = 8.0948(7) Å, *α* = 90°, *V* = 1602.8(2) Å^3^, *T* = 100(2) K, space group *Amm*2, *Z* = 2, 14889 reflections measured, 2152 independent reflections, *R*_*int*_ = 0.0344, *R*_*1*_ = 0.0279 (all data). CCDC 1017159.

### Cytotoxicity Assessment

Primary human foreskin-derived fibroblasts (HF) were cultured in Dulbecco’s modified Eagle’s medium (DMEM) (Cellgro, Manassas, VA) containing 4.5 g/ml glucose, 10% fetal bovine serum (SAFC, Lenexa, KS), 1 mM sodium pyruvate, 2 mM L-glutamine, and 100 U/ml penicillin-streptomycin (Cellgro) at 37 °C with 5% CO_2_^60^. HF cells were seeded in 12-well plates and allowed for 24 hours for confluency before treatment with **1**·EY at a final concentration from 0.1–500 µM. At 72 hours post treatment, bright-field images of living cells were captured using an inverted Evos-FL microscope (Thermo Fisher Scientific, Waltham, MA). After imaging, cell viability was determined by a trypan blue exclusion assay^51^ as previously described^61^ using a TC20 automated cell counter (Bio-Rad Laboratories, Hercules, CA). In brief, cells were harvested using 0.025% Trypsin-EDTA (Gibco, Thermo Fisher Scientific) diluted in phosphate-buffered saline and neutralized with equal volume of supplemented DMEM. 10 µl of cell suspension was mixed with an equal volume of trypan blue (Hyclone Laboratories, Logan, UT) and 10 µl of mixture was loaded immediately into the outer chamber of the counting slide. The slide was inserted into the slide slot of the cell counter, and percent of viable cells was calculated automatically.

### Computational Methods

All quantum chemical calculations in this study utilized the Gaussian 09 package^62^.

### Data Availability

All data generated or analyzed during this study are included in this published article and its Supplementary Information files.

## Electronic supplementary material

Supplementary Information
