# Supplementary material for: Highly selective and sensitive macrocycle-based dinuclear foldamer for fluorometric and colorimetric sensing of citrate in water
Source: Sci Rep. 2018 Jan 10;8:286. doi: 10.1038/s41598-017-18322-w (PMC5762659; doi:10.1038/s41598-017-18322-w)
Supplement: Supplementary file 1 — Supplementary Information [file 41598_2017_18322_MOESM1_ESM.pdf]

Supplementary Information

Highly selective and sensitive macrocycle-based dinuclear foldamer for fluorometric and colorimetric sensing of citrate in water

Md Mhahabubur Rhaman,[1] Mohammad H. Hasan,[2] Azmain Alamgir,[1] Lihua Xu,[3] Douglas R. Powell,[4] Bryan M. Wong*,[3] Ritesh Tandon*[2] and Md. Alamgir Hossain*[1]

[1] Department of Chemistry and Biochemistry, Jackson State University, Jackson, MS 39217, USA
[2] Department of Microbiology and Immunology, University of Mississippi Medical Center, Jackson, MS 39216, USA
[3]Department of Chemical & Environmental Engineering and Materials Science & Engineering Program, University of California-Riverside, Riverside, CA 92521, USA
[4]Department of Chemistry and Biochemistry, University of Oklahoma, Norman, OK 73019, USA

*Correspondence and requests for materials should be addressed to
M. A. H. (alamgir.hossain@jsums.edu), B. M. W. (bryan.wong@ucr.edu) or R. T. (rtandon@umc.edu)

Table of contents

Page

NMR and mass spectra of **L**....................S2

Packing diagram of **1'**....................S3

Glutamate titration....................S3

Phosphate titration....................S4

Adipate titration....................S4

Tartrate titration....................S5

Benzoate titration....................S5

Acetate titration....................S6

Selectivity test of citrate....................S6

Job's plot....................S7

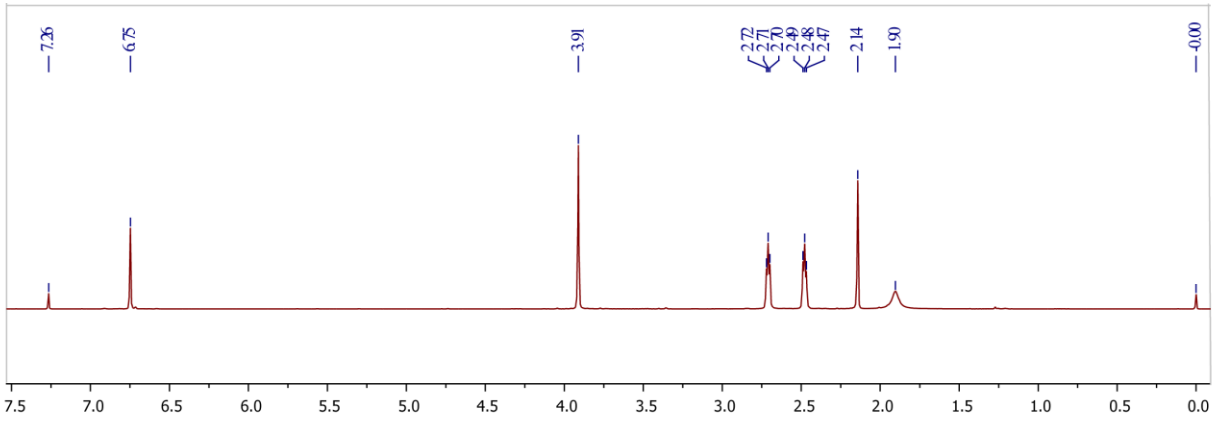

Figure S1. ^{1}H NMR of **L** in $CDCl_3$.

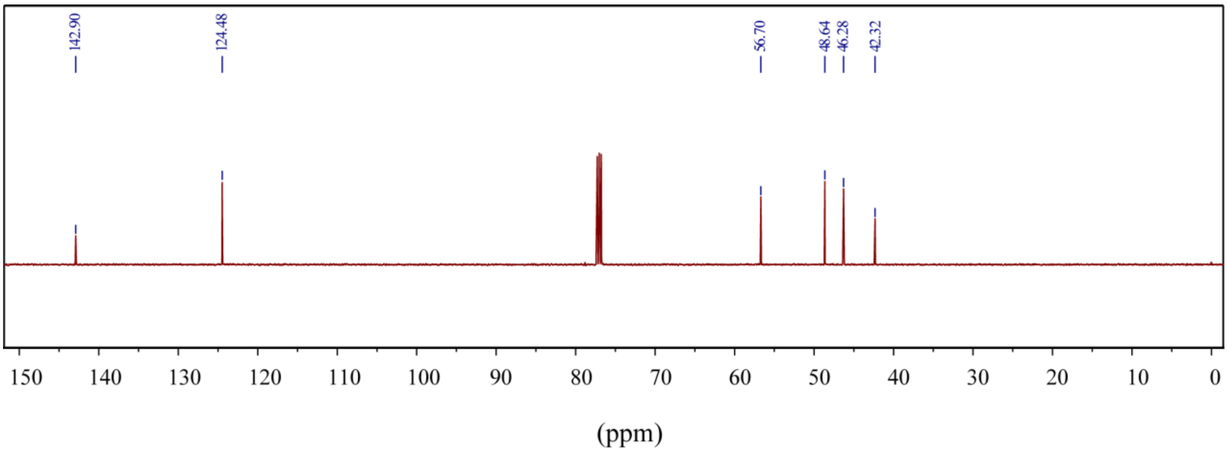

Figure S2. ^{13}C NMR of **L** in $CDCl_3$.

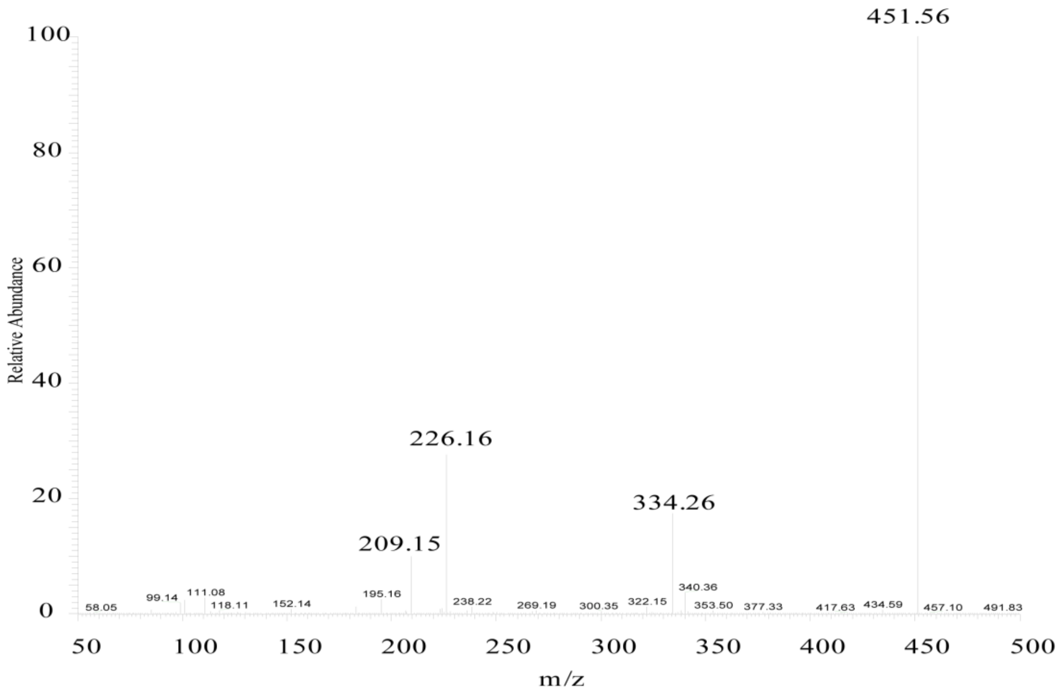

Figure S3. ESI-MS (+) spectra of **L**.

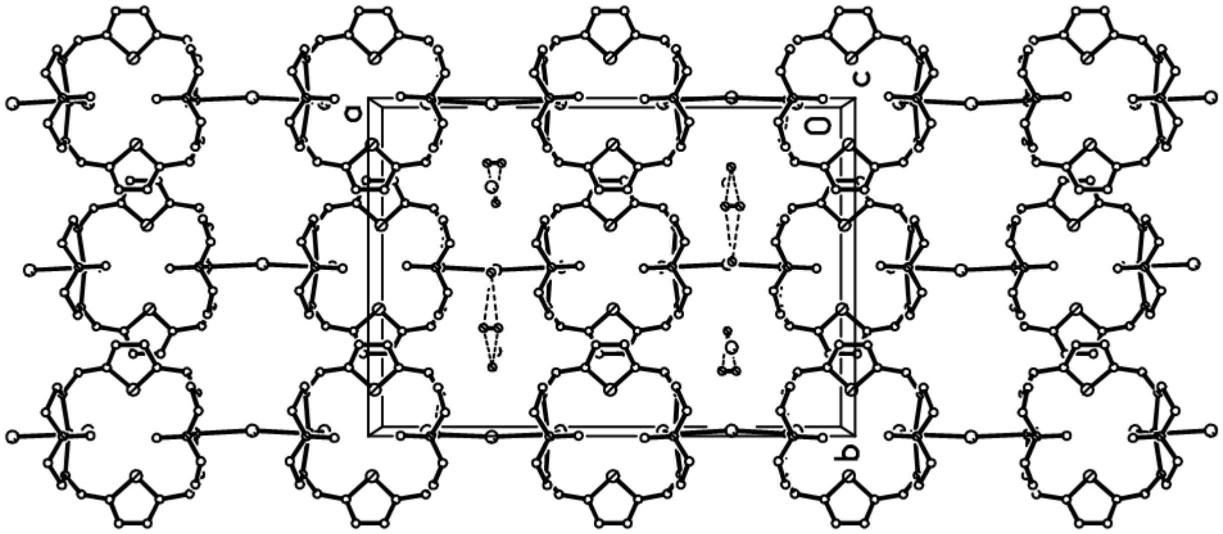

Figure S4. Packing diagram of **1'** showing an infinite polymer along the *a*-axis.

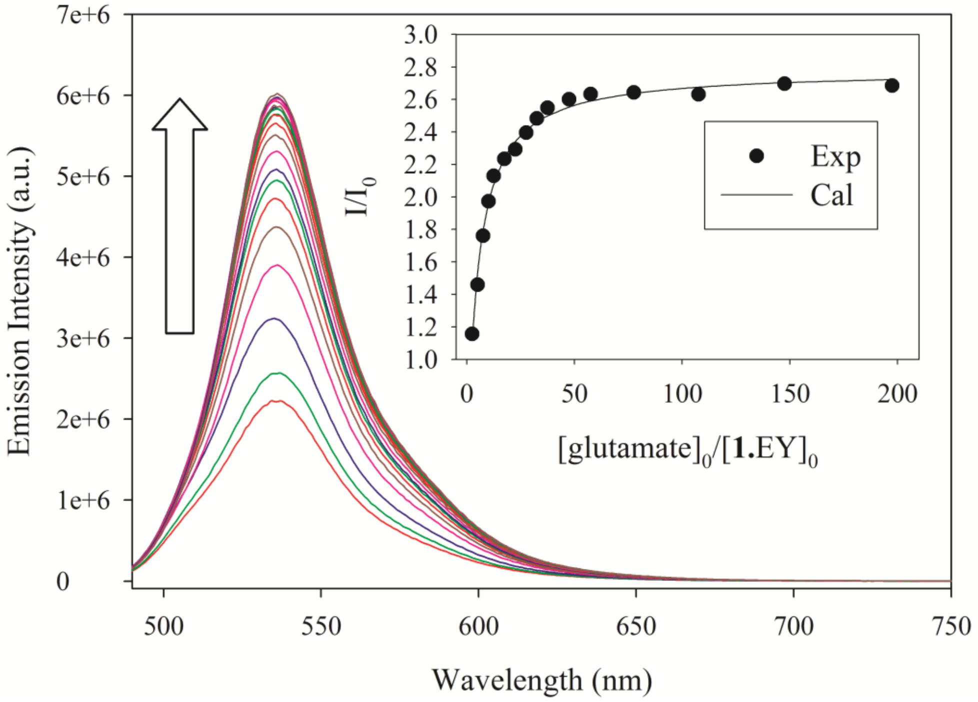

Figure S5. The enhancement of fluorescence intensity of [**1**·EY] (**1**/EY = 5:1) upon the addition of glutamate ([**1**·EY]$_0$ = 2 x 10^{-6} M, [glutamate]$_0$ = 2 x 10^{-3} M) in water buffered with 20 mM HEPES at pH 7.0. λ_{ex} = 470 nm, λ_{em} = 536 nm. The inset shows the titration plot of I/I$_o$ against [glutamate]$_0$/[**1**·EY]$_0$ at λ_{em} = 536 nm.

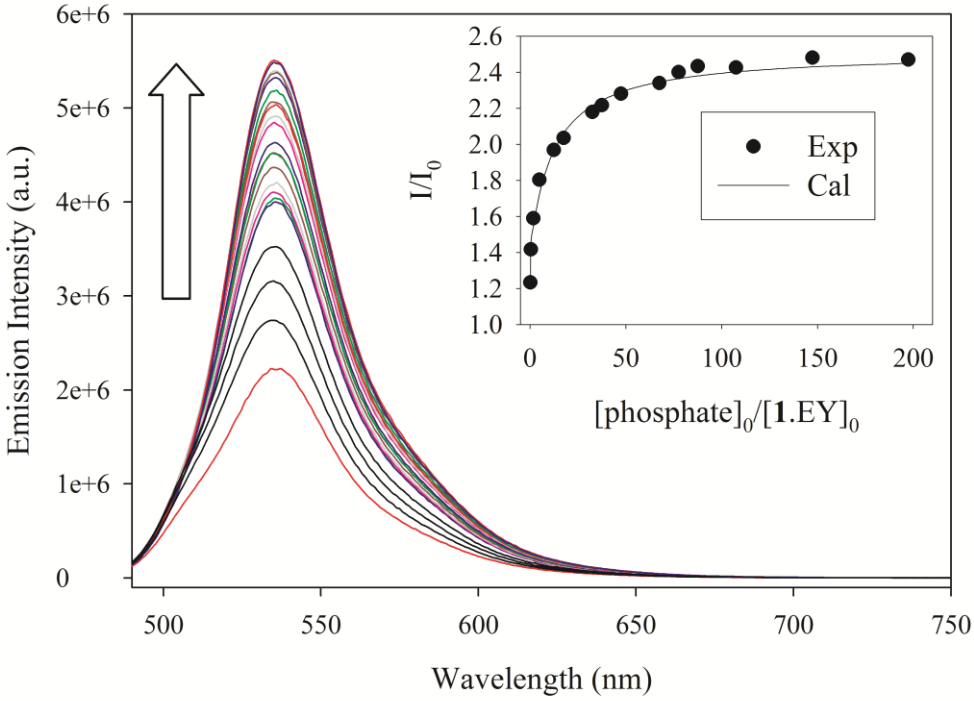

Figure S6. The enhancement of fluorescence intensity of [**1**·EY] (**1**/EY = 5:1) upon the addition of K_2HPO_4 ([**1**·EY]$_0$ = 2 x 10^{-6} M, [K_2HPO_4]$_0$ = 2 x 10^{-3} M) in water buffered with 20 mM HEPES at pH 7.0. λ_{ex} = 470 nm, λ_{em} = 536 nm. The inset shows the titration plot of I/I$_o$ against [K_2HPO_4]$_0$/[**1**·EY]$_0$ at λ_{em} = 536 nm.

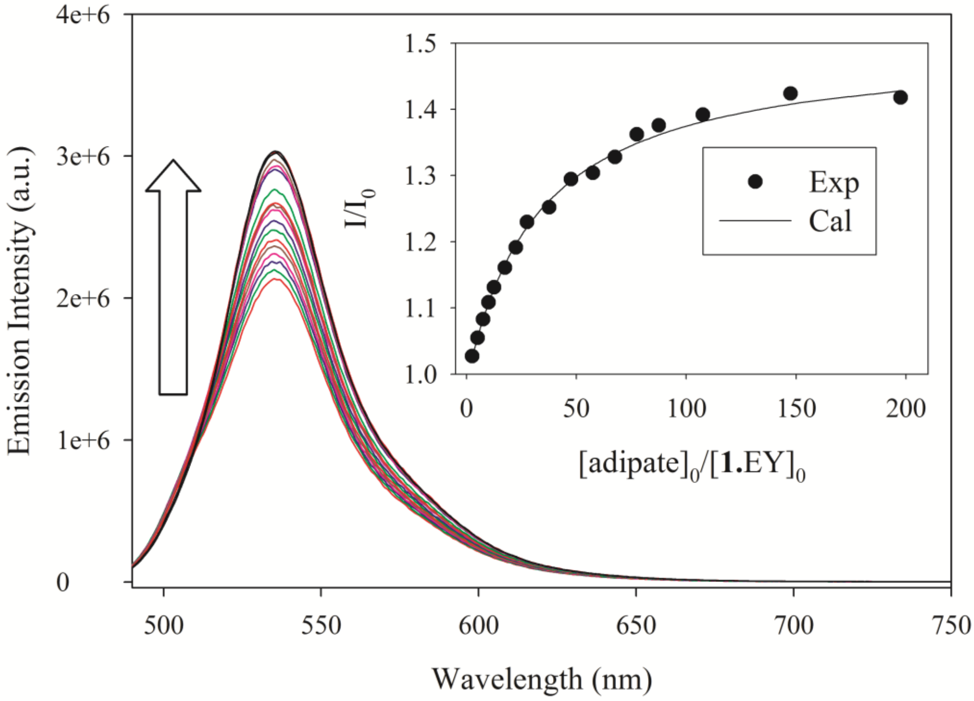

Figure S7. The enhancement of fluorescence intensity of [**1**·EY] (**1**/EY = 5:1) upon the addition of adipate ([**1**·EY]$_0$ = 2 x 10^{-6} M, [adipate]$_0$ = 2 x 10^{-3} M) in water buffered with 20 mM HEPES at pH 7.0. λ_{ex} = 470 nm, λ_{em} = 536 nm. The inset shows the titration plot of I/I$_0$ against [adipate]$_0$/[**1**·EY]$_0$ at λ_{em} = 536 nm.

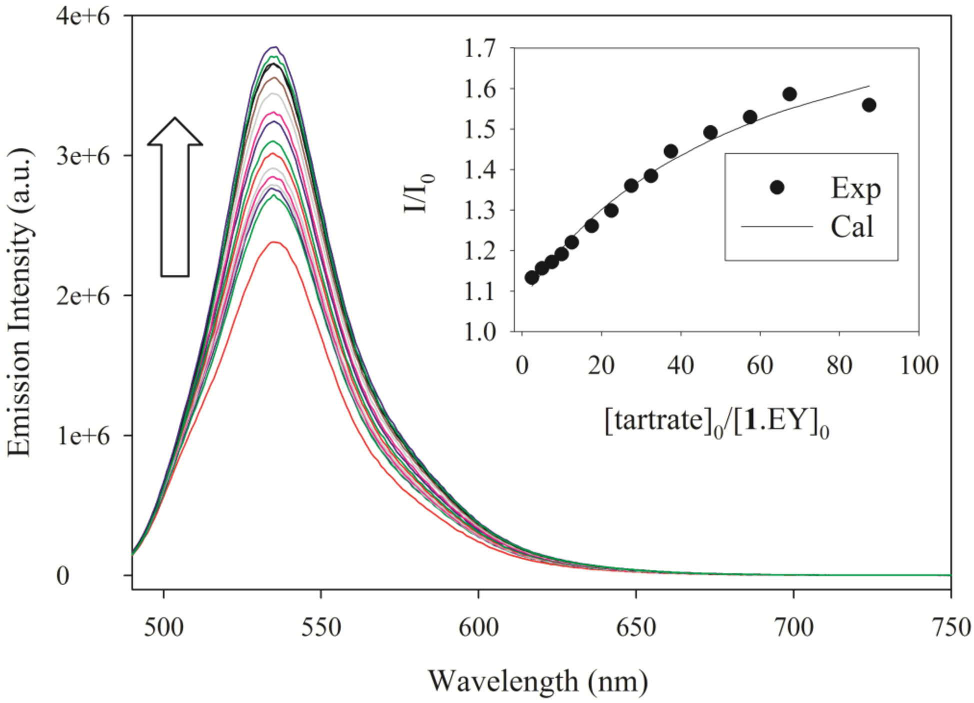

Figure S8. The enhancement of fluorescence intensity of [**1**·EY] (**1**/EY = 5:1) upon the addition of tartrate ([**1**·EY]$_0$ = 2 x 10^{-6} M, [tartrate]$_0$ = 2 x 10^{-3} M) in water buffered with 20 mM HEPES at pH 7.0. λ_{ex} = 470 nm, λ_{em} = 536 nm. The inset shows the titration plot of I/I$_0$ against [tartrate]$_0$/[**1**·EY]$_0$ at λ_{em} = 536 nm.

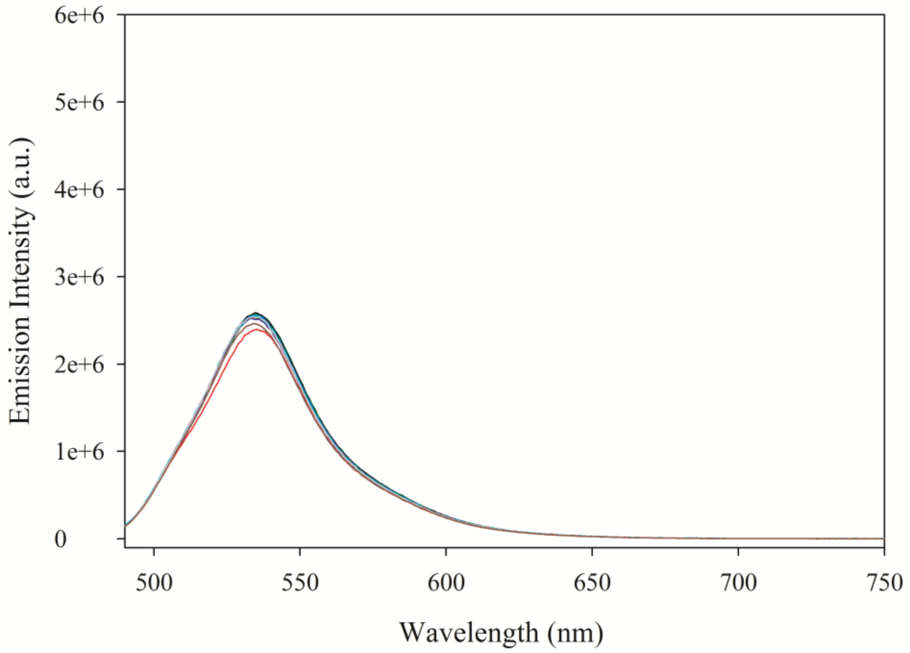

Figure S9. The fluorescence intensity of [**1**·EY] (**1**/EY = 5:1) upon the addition of benzoate ([**1**·EY]$_0$ = 2 x 10^{-6} M, [benzoate]$_0$ = 2 x 10^{-3} M) in water buffered with 20 mM HEPES at pH 7.0. λ_{ex} = 470 nm, λ_{em} = 536 nm, showing insignificant change in fluorescence intensity.

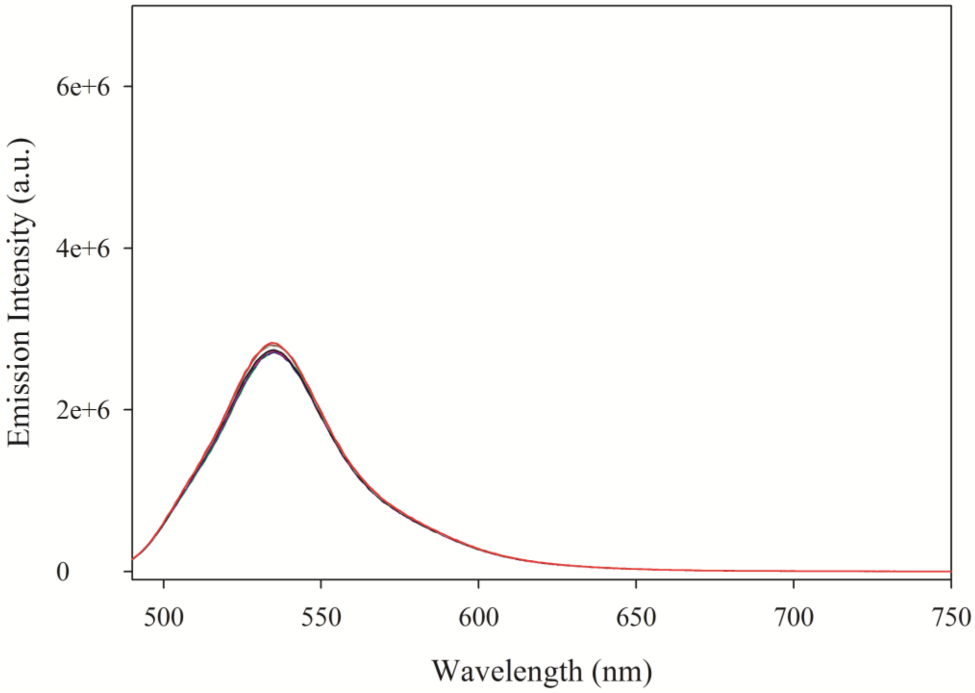

Figure S10. The fluorescence intensity of [**1**·EY] (**1**/EY = 5:1) upon the addition of acetate ($[\mathbf{1}\cdot EY]_0 = 2 \times 10^{-6}$ M, $[acetate]_0 = 2 \times 10^{-3}$ M) in water buffered with 20 mM HEPES at pH 7.0. λ_{ex} = 470 nm, λ_{em} = 536 nm, showing insignificant change in fluorescence intensity.

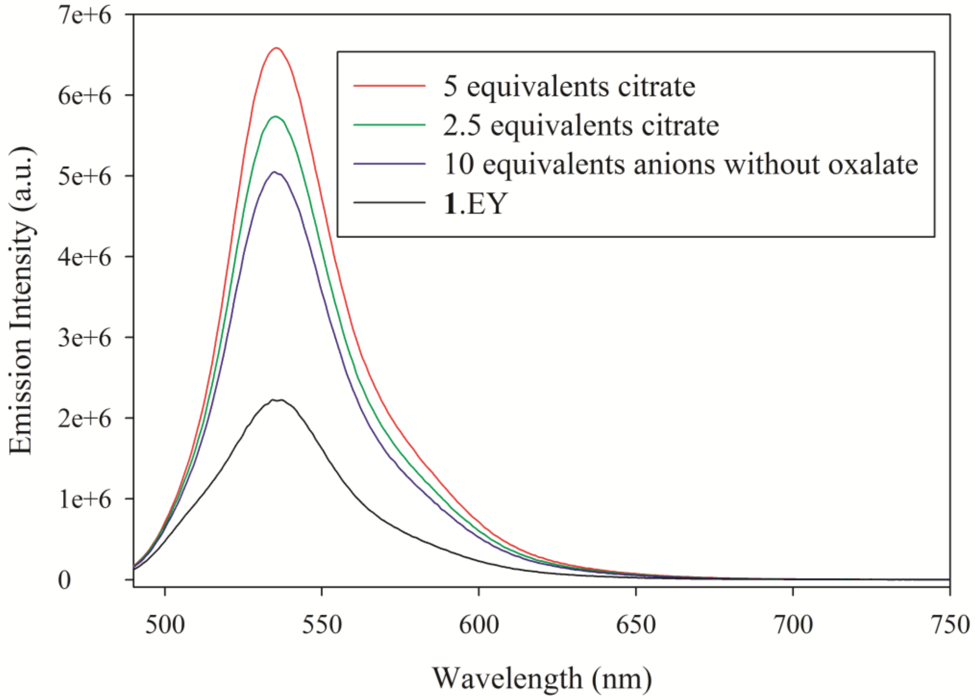

Figure S11. The selectivity of citrate in presence of 10 equivalents of other anions without oxalate, λ_{ex} = 470 nm, λ_{em} = 536 nm, d_{ex} = 2, d_{em} = 3.

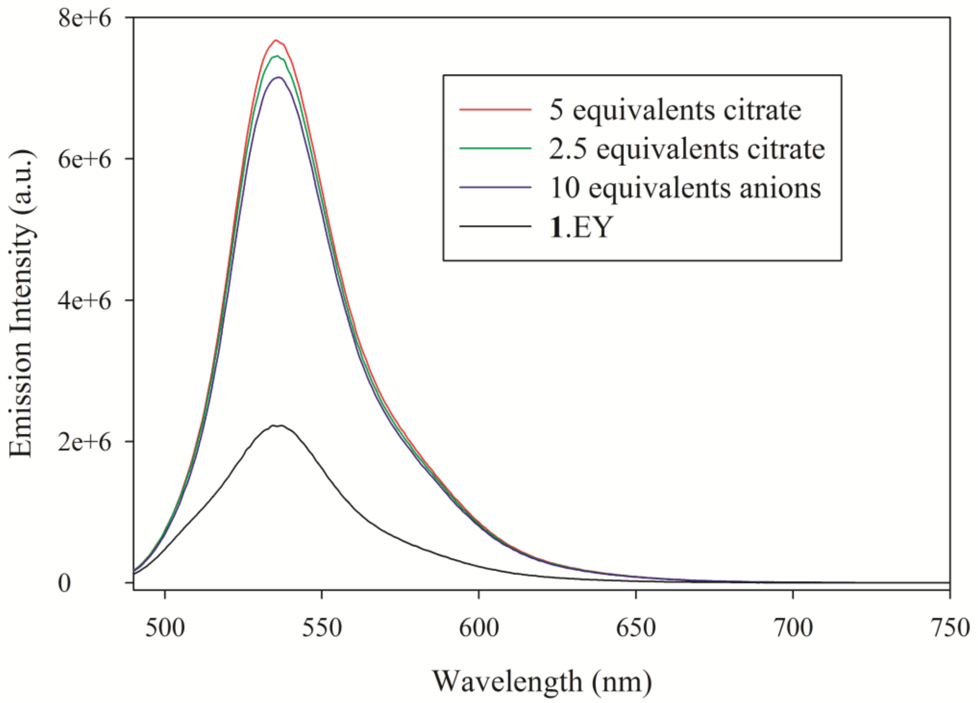

Figure S12. The selectivity of citrate in presence of 10 equivalents of other anions, λ_{ex} = 470 nm, λ_{em} = 536 nm, d_{ex} = 2, d_{em} = 3.

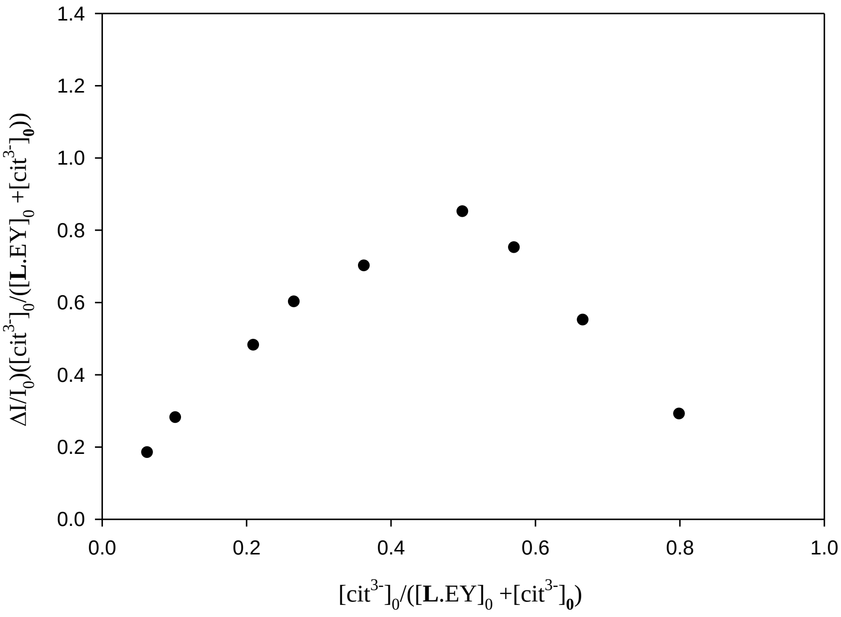

Figure S13. Job's plot for the formation of the citrate complex with **1**.EY. The data was obtained from the titration of **1**.EY with citrate as shown in Figure 4 in the main manuscript.
